# Visuo-spatial cueing in children with differential reading and spelling profiles

**DOI:** 10.1371/journal.pone.0180358

**Published:** 2017-07-07

**Authors:** Chiara Banfi, Ferenc Kemény, Melanie Gangl, Gerd Schulte-Körne, Kristina Moll, Karin Landerl

**Affiliations:** 1 Institute of Psychology, University of Graz, Graz, Austria; 2 Department of Child and Adolescent Psychiatry, Psychosomatics, and Psychotherapy, Ludwig-Maximilian University, Munich, Germany; Katholieke Universiteit Leuven, BELGIUM

## Abstract

Dyslexia has been claimed to be causally related to deficits in visuo-spatial attention. In particular, inefficient shifting of visual attention during spatial cueing paradigms is assumed to be associated with problems in graphemic parsing during sublexical reading. The current study investigated visuo-spatial attention performance in an exogenous cueing paradigm in a large sample (*N* = 191) of third and fourth graders with different reading and spelling profiles (controls, isolated reading deficit, isolated spelling deficit, combined deficit in reading and spelling). Once individual variability in reaction times was taken into account by means of *z*-transformation, a cueing deficit (i.e. no significant difference between valid and invalid trials) was found for children with combined deficits in reading and spelling. However, poor readers without spelling problems showed a cueing effect comparable to controls, but exhibited a particularly strong right-over-left advantage (position effect). Isolated poor spellers showed a significant cueing effect, but no position effect. While we replicated earlier findings of a reduced cueing effect among poor nonword readers (indicating deficits in sublexical processing), we also found a reduced cueing effect among children with particularly poor orthographic spelling (indicating deficits in lexical processing). Thus, earlier claims of a specific association with nonword reading could not be confirmed. Controlling for ADHD-symptoms reported in a parental questionnaire did not impact on the statistical analysis, indicating that cueing deficits are not caused by more general attentional limitations. Between 31 and 48% of participants in the three reading and/or spelling deficit groups as well as 32% of the control group showed reduced spatial cueing. These findings indicate a significant, but moderate association between certain aspects of visuo-spatial attention and subcomponents of written language processing, the causal status of which is yet unclear.

## Introduction

Developmental dyslexia is a neurodevelopmental disorder characterized by difficulties with accurate or fluent reading and/or spelling problems, despite adequate instruction, intelligence and intact sensory abilities. It affects about 7% of the population, depending on the cutoff criteria used for defining reading problems [1]. In the last decades, plenty of studies investigated the cognitive and neuropsychological deficits underlying dyslexia. Impairments within the domains of phonological awareness (PA) and rapid automatized naming (RAN) were consistently shown to be strongly associated with dyslexia [2,3] and represent its most significant predictors, with a different load across orthographies [4]. For example, a recent study by [5] showed that about 30% of children with reading problems showed deficits in each of these two domains at school entry, while only about 13% of children with typical development performed poorly in these tasks. Interestingly, such findings indicate that the causes of poor reading outcome cannot be explained by one single deficit, but are multifactorial and probabilistic, rather than deterministic [5,6].

Even though the strength of the association is less clear compared to PA and RAN, dyslexia has also been associated with deficits in visuo-spatial attention and more specifically in orienting spatial attention in exogenous spatial cueing paradigms [7–12]. In this task originally developed by Posner [13] a cue is briefly presented in the periphery on the left or the right of a central fixation cross. It is shortly followed by the appearance of the target, which is either spatially consistent with the cue (valid trials) or not (invalid trials) [14]. Valid cues orient attention to the target location, triggering shorter response times (RTs) because of enhanced processing at the attended location; Invalid cues exert slower responses because the cued location needs to be suppressed in order to respond accurately [10]. The cueing effect refers to the advantage of valid over invalid trials. If present, it indicates the amount of attentional filtering, with attentional focus on the cued location. An unduly small or large cueing effect suggests that attentional filtering operates less efficiently, indicating that the attentional focus may be too broad or too narrow, respectively. Differently from the exogenous task, in endogenous visuo-spatial cueing paradigms the cue is presented in the center of the visual field at longer SOAs and is usually predictive of the target, which appears in the periphery. While endogenous paradigms require top-down orienting, and attention is voluntarily shifted to the attended location through eye-movements, exogenous tasks are taken to indicate bottom-up attention orienting [14]. Interestingly, a number of studies reported reduced or absent cueing effects in children and adults with dyslexia in exogenous as well as endogenous spatial cueing paradigms [7–12, 15–17], cued visual search [18–20], cued texture detection [21], and cued coherent motion detection [22].

In addition to the impairment in orienting attention, a number of studies found that individuals with dyslexia show an asymmetrical distribution of their visual attention, with better performance on targets in the right than the left visual field, while controls performed equally well on both sides [7,10,17,23–26]. This position effect was initially attributed to a left-sided minineglect [26], but was later explained in terms of a specific deficit of the right attentional inhibitory mechanism [10], or, more recently, described as weaker attention in the left visual field [23]. For visuo-spatial cueing paradigms, however, the evidence on positional asymmetries is mixed: [9] found a position effect in an endogenous, but not an exogenous task. Surprisingly, this position effect was equally large for dyslexic and typical readers. Instead, in a mixed endogenous/exogenous paradigm [10] reported an interaction of cueing and position for dyslexic readers with poor nonword reading skills only: These children showed a right-over-left advantage for invalid, but not for valid trials. A specific position effect for invalid trails in dyslexia was confirmed by [17]. However, their paradigm included endogenous stimuli only and they did not differentiate between good and poor nonword readers in their adult dyslexia sample. Up to date, these findings do not allow a clear explanation of the mechanisms underlying spatial asymmetries in cueing paradigms.

Efficient performance on the spatial cueing paradigm requires to quickly orient attention towards the target and to adequately adjust the attentional window according to task demands [27]. [9,11] argued that individuals with dyslexia have difficulties shifting attention to cues, thus sustaining the Sluggish Attentional Shifting theory (SAS) proposed by [28]. According to the SAS account, individuals with dyslexia experience very slow attentional engagement, due to sluggish attentional capture, as well as problems in attention dis-engagement because of prolonged attentional dwell. More recently, [21] suggested to differentiate between the concepts of focusing and orienting. In their view, the difficulties of dyslexic individuals in visuo-spatial paradigms derive from a reduced or less powerful spotlight of attention, i.e. an impairment in focusing, rather than from problems in shifting attention to cues.

The exact mechanisms underlying the association between visual attention and written language processing are also not very well understood. Reading models suggest the involvement of focused visuo-spatial attention in the process of graphemic parsing during assembly of phonology from print [29–31]. Focused visuo-spatial attention allows efficient serial scanning of the letter string, thus enabling visual processing in terms of high speed and spatial sensitivity. At the same time, focused visual attention might reduce interference between neighboring stimuli, also known as visual crowding [32,33]. Given that sublexical decoding is generally assumed to provide an important self-teaching mechanism for the build-up of written word representations [34], [29] propose in their model that visuo-spatial attention may also impact on orthographic processing by providing precise within-word positional information of letter identities. Hence, a visuo-spatial attention deficit hampering sublexical reading might affect orthographic spelling as well.

Associations of visuo-spatial cueing and serial decoding as required in nonword reading have repeatedly been shown: [10–12] found a selective absence of a cueing effect in dyslexic individuals with poor nonword reading accuracy. Dyslexics with nonword reading accuracy within the normal range (but low reading fluency) displayed a cueing effect comparable to controls. However, replication of these findings is still needed as samples in previous studies comprised a broad age range, and were rather small with only 10 to 18 dyslexic individuals per group. Furthermore, about half of this sample showed rather high nonword reading accuracy (75 to 80% correct), so their nonword reading skills were only moderately compromised in the highly transparent Italian orthography.

While poor performance in dyslexic compared to typical readers can provide first evidence for an association between visual attention processing and dyslexia, it cannot establish causality [35]. In order to support causal claims, [11] provided evidence for a reduced cueing effect in dyslexic readers with low nonword reading accuracy compared to younger typical readers matched on word reading efficiency. The cueing effect of dyslexic readers with relatively high nonword reading accuracy was not different from reading as well as chronological age matched children. [36] showed that visuo-spatial attention performance in kindergarten was a significant predictor of reading in Grades 1 and 2. However, this study did not control for early reading skills in kindergarten so that it is unclear whether better kindergarten cueing performance was mostly evident in those children who already had early reading experience. Perhaps the strongest evidence in support of causal links are training designs and indeed, two studies [37,38] reported improved nonword and text (though not word) reading skills in groups of ten and eleven dyslexic children after nine sessions of playing action video games which were specifically selected to stimulate the dorsal or “action” stream of visual processing, thus supposed to address visuo-spatial attentional skills.

Less evidence is available on associations of visuo-spatial attention with lexico-orthographic processing. Word reading is perhaps not such a good test case for lexical processing as words can mostly be read lexically as well as sublexically, particularly in orthographies that are phonologically more transparent than English. Instead, spelling is more revealing, as in most orthographies correct word spelling requires exact retrieval of a letter sequence including orthographic markers that are not specified by a simple phoneme-grapheme translation strategy. Interestingly, [39,40] could identify a considerable percentage of children learning to read in German who showed marked dissociations between nonword (as well as word) reading efficiency on the one hand, and orthographic spelling on the other. The current study is based on a research project more closely investigating such dissociations of deficits in reading and spelling in German orthography, which is consistent in the reading, but inconsistent in the spelling direction. We tested visuo-spatial cueing in children with isolated reading deficits (and age adequate spelling) as well as children with isolated spelling deficits (and age adequate reading). These data will help to assess to what extent visuo-spatial processing is associated with lexical and sublexical written language processing.

One particular concern for any causal claim on visuo-spatial deficits in dyslexia is that this learning disorder is frequently comorbid with broader attentional problems like ADHD [41]. Thus, it needs to be established that the findings on impaired attentional cueing in dyslexia are not due to unidentified comorbid attentional deficits. In the studies summarized above, children with ADHD were usually excluded from participation, however, this may not be sufficient as not having a clinical diagnosis of ADHD is by no means equal to having an age-adequate attentional profile—children may only just have missed diagnostic criteria. Indeed, [42] showed that many dyslexic individuals have attentional impairments in the subclinical range, which may not always be detected in small samples. Furthermore, asymmetric performance depending on position are not only reported for individuals with dyslexia but also for individuals with high ADHD scores [43], thus suggesting that general attention capabilities might be related to the position effects in cueing paradigms.

Obviously, such deficits may easily extend to attentional problems in the visuo-spatial domain. In the current study, we were able to assess a relatively large sample of almost 200 children with an exogenous cueing paradigm and we asked their parents to answer a standardized ADHD-questionnaire. These data will allow us to test whether the proposed cueing deficit is indeed specific to dyslexia.

Finally, we aimed to investigate the prevalence of cueing deficits among typically developing readers. The control samples reported in the studies summarized above were small and therefore not informative to this point. As [44] pointed out, if even quite low percentages of a certain deficit in small control groups are extrapolated for the general population, this would indicate that a considerable number of individuals with visuo-spatial deficits are well able to develop adequate (nonword) reading skills. The present sample included a relatively large control group of 66 children with typical development in reading as well as spelling. It will be interesting to see how many of these children show evidence for visuo-attentional impairments.

In summary, the present study addressed the following research questions:

1. If automatic orienting of visuo-spatial attention is involved in reading, we expect poor readers to show reduced cueing effects, and possibly increased position effects in an exogenous cueing paradigm.
2. More specifically, if visuo-spatial attention is related to graphemic parsing during sequential decoding [10,30], children with poor nonword reading skills should show impaired visual attention, irrespective of their spelling skills. Deficits in orthographic spelling that are not accompanied by deficits in sequential decoding should not be associated with deficient visuo-spatial attention.
3. If deficits in visual attention are specific to reading failure, they should remain after controlling for individual differences in ADHD-symptoms.
4. We expect the rate of occurrence of visuo-spatial deficits to be relatively low among children with typically developing reading skills and clearly higher among children with deficits in written language processing.

## Materials and methods

### Participants

The study was approved by the ethics committee of the University of Graz and by the institutional review board of Medical Faculty of the University Hospital Munich. It was performed in accordance with the latest version of the Declaration of Helsinki and in compliance with national legislation. Written informed consent was obtained on behalf of the children from their parents. Children were selected based on an extensive classroom screening with 4123 children at the end of 3^rd^ Grade or beginning of 4^th^ Grade, which was carried out in two collaborating sites, Munich (Germany) and Graz (Austria). To start with, standardized classroom tests of sentence reading fluency (SLS 2–9: [45]) and spelling were given (DRT 3: [46]). Classroom assessments (maximum classroom size: 25 children) were always carried out by two experimenters, with one presenting the instruction and the other one observing whether all children followed instructions attentively and providing individual support if needed.

Children who performed at or below percentile 20 in sentence reading fluency and/or spelling were also administered a standardized one-minute word and nonword reading speed test (SLRT-II: [47]), either in school or in the lab. Only children who also performed below percentile 20 on one of these subtests and below average on the other were identified as poor readers. Age-adequate performance was defined as percentiles between 25 and 85 on the mean of the three reading measures (sentence, word and pseudoword reading) as well as on spelling.

On basis of the screening procedure, 207 participants were recruited. Because of outlier performance on the spatial cueing paradigm (details see below), the data of 16 children were not further analyzed. The final sample consisted of 191 children belonging to four groups: controls (C; *n* = 66), isolated reading deficit with age-adequate spelling (RD; *n* = 28), isolated spelling deficit with age-adequate reading (SD; *n* = 45), and combined reading and spelling deficit (RSD; *n* = 52). Note that one child of the RD group and one child of the RSD group had not participated in the classroom screening but received the SLRT-II and an age adequate standardized spelling test (DRT 4: [48]) during an individual assessment.

All children had German as their first language, a non-verbal IQ ≥ 85 (CFT 20-R: [49]), normal or corrected-to-normal vision, no identified sensory or neurological deficits, no clinical ADHD diagnosis as well as an above-threshold score on a parental questionnaire for attention deficits (FBB-ADHS, DISYPS-II: [50]).

### Tasks

The tasks described in this paper were part of a larger task battery comprising an initial screening in school including the classroom measures of sentence reading and spelling and the nonverbal IQ test. The individually administered word and nonword reading test was given in school or in the lab. All other tasks were carried out as part of three to four individual assessments in our labs, each of which lasted between 90 and 120 minutes. The tasks described here were usually carried out during the first and second assessments, which took place about 2 to 12 weeks after the classroom screening.

#### Reading

In the classroom-administered standardized reading speed task (SLS 2–9: [45], parallel test reliability is .95 for Grade 2 and .87 for Grade 8), children were asked to silently read single-line-long sentences with simple semantic and syntactic structure (e.g., “Trees can speak”); they had to mark each sentence as right or wrong by circling a check mark or a cross at the end of the line. The task was terminated after three minutes. The raw score was the number of correctly marked sentences.

In the individually administered one-minute reading speed task (SLRT-II: [47], parallel test reliability is .94 for words and .90 for nonwords), children were instructed to read aloud a word and a nonword list as fast as possible without making errors. The number of correctly read items within one minute was taken as raw score.

#### Spelling

The standardized classroom spelling task (DRT 3: [46]; split-half reliability is .95 according to the manual) comprised 44 words that had to be written into sentence frames. The experimenter dictated each word, then read out the full sentence and then repeated the word again. The number of correct word spellings was scored.

#### Nonverbal IQ

The first part of the German version of the Culture Fair Intelligence Test (CFT 20-R: [49]; test reliability = .92 according to manual) was given as an estimate of nonverbal IQ. Its four subtests comprised Series, Classification, Matrices and Topology.

#### Vocabulary

Was assessed by the vocabulary subtest of the German version of the Wechsler Intelligence Scale for Children (WISC-IV: [51]).

#### Verbal short-term and working memory

Were investigated by the Digit Span subtest of the German version of the Wechsler Intelligence Scale for Children (WISC-IV: [51]).

#### Speed of processing

Was investigated by the Symbol Search subtest of the German version of the Wechsler Intelligence Scale for Children (WISC-IV: [51]).

#### ADHD-rating

Parents were asked to answer a standardized questionnaire (DISYPS-II: [50]) which consists of 20 items with a 4-point rating scale investigating symptoms of inattention (9 items), hyperactivity (7 items) and impulsivity (4 items). A high score on the questionnaire is indicative of high ADHD symptoms.

#### Phonological awareness (PA)

Was assessed by means of a computerized phoneme deletion task running on Presentation 16.3 (Neurobehavioral Systems, Inc., Berkeley, CA, USA). The task consisted of four practice trials and 25 test trials (20 mono- and 5 disyllabic nonwords) which were presented via headphones. Children were asked to repeat each nonword first and then to pronounce it without a specified phoneme (e.g., “/folt/ without /t/”). Any nonword that children could not pronounce correctly was played again up to two times. Items that were still not repeated correctly (no more than 3 per participant) were excluded from analysis. The ratio of correct responses to the total number of responses was taken into account. Cronbach’s alpha was .76.

#### Rapid automatized naming (RAN)

Children were asked to name a matrix of 40 digits as quickly and accurately as possible. Five digits (8, 3, 5, 2, 9) were presented in five columns and eight lines. The order of the items was randomized and each item was presented once in each line. The experimenter recorded the time needed to name the full item set as well as any occurring errors. The raw score was the number of digits named correctly per second.

#### Cueing task. apparatus

The experimental cueing paradigm was run inside a dimly lit room and was controlled with Experiment Builder software (RS Research, version 1.10.1241). Children were seated in front of a computer screen (Graz: 120-Hz refresh rate, 1024 x 768 pixels; Munich: 120-Hz refresh rate, 1280 x 960 pixels) at a viewing distance of about 65 cm. To ensure that children looked at the central fixation cross, eye movements were monitored using an EyeLink 1000 tower mount eye tracker in Graz and an EyeLink 1000 Plus desktop mount eye tracker in Munich (SR Research, Toronto, Canada). Eye-movement data were not further analyzed. Note that, since monitor sizes differed between the two labs, the dimensions of stimuli were changed in order to keep constant the visual angle subtended by the stimuli and their relative distance.

#### Stimuli

Items were displayed in black on a white background. The experiment comprised three conditions with 16 items each. On valid trials the cue appeared in the same position as the target, on invalid trials the cue appeared on the opposite side of the target. The left/right position of cues and targets was balanced. Finally, on catch trials only the cue appeared, without a following target. In order to avoid a response-bias deriving from the frustration after a catch trial, we introduced fake items after each catch trial. Fake items were balanced for condition (valid vs. invalid) and position and were not considered in the analyses. Children received oral instructions accompanied by visual examples as well as eight practice trials. Experimental trials were pseudo-randomized with the restriction that no more than two items of the same condition appeared in immediate succession. Four equivalent pseudo-randomized versions of the experiment were prepared and randomly assigned to the participants. Due to experimental error, three of the pseudo-randomized versions were not implemented as planned in one of the participating labs. As a consequence, for 87 children (29 C, 15 RD, 16 SD and 27 RSD) the number of items per position and cueing condition varied between 5 and 13 (instead of 8). Note that in the final analysis, item numbers varied between children anyway, as only correct responses were included. To check for possible differences in performance between children who received the balanced vs. unbalanced task versions, independent-samples t-tests were run on the cueing index and the position index (see further for details on those indexes). There were no significant differences: Cueing index *t*(189) = -1.00, *p* = .32; position index *t*(189) = .96, *p* = .34.

#### Procedure

**Fig 1:**
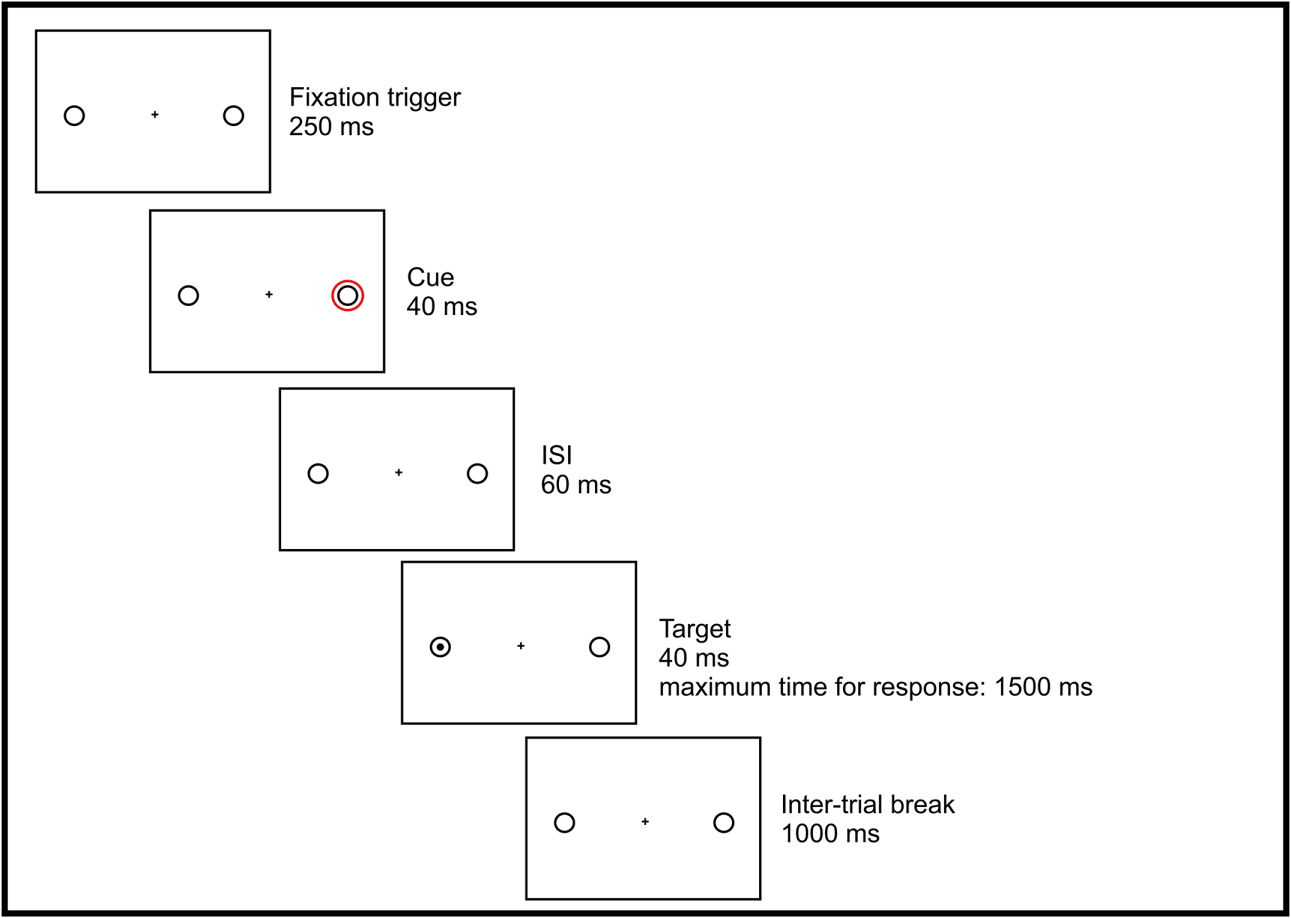
A trial of the visuo-spatial cueing paradigm.

The cueing experiment started with a nine-point calibration of the eye-tracking system, which was used to ensure that participants looked at the fixation cross at the beginning of each item. As Fig 1 shows, each trial began with a slide showing a central fixation cross and two black circles, which subtended 2.5° of visual angle and were presented peripherally at 8° of eccentricity. Immediately afterwards a red circle (the cue) appeared around one of the two black circles, which was started by a fixation trigger, importantly, it showed up only if children looked at the fixation cross. In case of a fixation trigger failure, children underwent a new calibration procedure and started the experiment from the point where it was interrupted. The red circle lasted 40ms and was followed by a 60ms display, showing again a central fixation cross and two black circles (SOA = 100ms). Next, a black point (the target) appeared within one of the two circles for 40ms. Children were instructed to press the space bar as soon as they saw the target appearing and not to press it if there was no target. The time-window allowed to respond was set to 1500ms. After a blank screen appearing for 1000ms, a new trial began.

#### Data preprocessing

Only correct responses in the valid and invalid conditions were analyzed. Data loss due to errors of omissions was 6.5% of the overall dataset (6624 trials overall, 6194 accurate trials). Internal consistency was assessed by means of the split-half correlation on RTs between odd and even correct trials, which yielded a high and significant Pearson correlation coefficient, *r*(205) = .869, *p* < .001.

RTs equal to or lower than 150ms were considered anticipatory whereas responses above 2.5 standard deviations from the individual mean were treated as outliers and discarded from the statistical analysis. This procedure removed 168 trials, corresponding to the 2.5% of the initial dataset. After excluding outliers, exploratory data inspection showed that one child still had an unrealistically high zRT-score of 1.45 in one of the conditions (valid left), which was caused by one single outlier response representing a RT of 3 *SD*s above the overall mean of the participant after outlier removal. This outlier RT was thus removed.

Fifteen participants (6 C, 3 RD, 3 SD, 3 RSD) were excluded due to overall accuracy rates below 75% or less than 3 correct responses per condition. One child from the SD group was further removed because his mean RT was more than 4 standard deviations above the overall mean RT of the entire sample.

## Results

### Literacy and cognitive measures

Table 1 summarizes the descriptive, literacy and cognitive variables for the 191 participants included in the analysis. The literacy measures were our selection criteria, therefore, group differences were to be expected: the RD and RSD groups showed lower performance than controls and the SD group on sentence, word and nonword reading, while the SD and RSD groups had lower spelling scores than controls and the RD group. The RD group showed age-adequate spelling skills, but note that their spelling was still slightly and significantly lower than controls´ spelling performance. The SD group showed age-adequate reading performance, which did not differ from controls. The RSD group did not differ from the RD group in any of the reading tasks and they also did not differ from the SD group in spelling, indicating that children in the RSD group were not more severely impaired than children with an isolated reading or spelling deficit.

**Table 1:** Means (*M*) and standard deviations (*SD*) for age, literacy and cognitive measures in the four groups.

	C *n* = 66			RD *n* = 28			SD *n* = 45			RSD *n* = 52				
		
	*M*		*SD*	*M*		*SD*	*M*		*SD*	*M*		*SD*	*F*	*p*
Age (months)	112.35		4.34	113.79		5.58	114.53		6.58	112.98		5.93	.22	0.21
Sentence reading (SLS):														
percentile	52.43	2,4	14.36	11.32	1,3	4.87	49.16	2,4	15.80	9.64	1,3	8.80	162.75	<.001
raw score	36.11	2,4	3.76	22.52	1,3	3.14	35.02	2,4	4.86	20.39	1,3	4.65	181.49	<.001
Word reading (SLRT-II):														
percentile	51.21	2,4	16.18	14.70	1,3	8.48	45.23	2,4	17.84	8.92	1,3	7.43	117.24	<.001
raw score	64.15	2,4	11.04	39.25	1,3	6.07	60.3	2,4	12.08	33.29	1,3	7.87	118.22	<.001
Nonword reading (SLRT-II):														
percentile	52.59	2,4	18.10	13.32	1,3	6.41	47.88	2,4	22.23	12.69	1,3	9.47	84.70	<.001
raw score	39.18	2,4	5.37	25.68	1,3	3.40	38.16	2,4	7.45	24.46	1,3	5.46	90.89	<.001
Spelling (DRT-3):														
percentile	54.45	2,3,4	13.04	43.48	1,3,4	13.91	13.22	1,2	4.95	10.33	1,2	6.86	244.19	<.001
raw score	26.09	2,3,4	3.81	22.93	1,3,4	4.10	11.93	1,2,4	2.49	9.84	1,2,3	4.34	235.65	<.001
Nonverbal IQ (CFT-20)	107.12		11.08	109.71		15.95	103.20		10.50	105.50		11.95	1.89	0.13
WISC-IV (standard score)														
vocabulary	12.33		3.02	13.14		3.00	11.36		3.41	12.08		3.28	1.95	0.13
digit span	10.15		2.21	10.68		2.00	9.93		2.35	9.69		2.45	1.23	0.31
symbol search	12.03	3,4	2.24	10.89		1.59	10.76	1	2.67	10.81	1	2.00	4.17	0.01
Phonological awareness (% correct)	0.82	4	0.12	0.76	4	0.12	0.77	4	0.14	0.64	1,2,3	0.19	14.51	<.001
RAN digits/s	2.16	2,4	0.43	1.78	1	0.27	2.02	4	0.41	1.74	1,3	0.33	14.14	<.001
ADHD questionnaire	0.41	3	0.31	0.48		0.32	0.66	1	0.34	0.53		0.36	5.11	0.002

*Note*. Subscripts indicate significant differences on post-hoc analyses with Bonferroni correction for multiple comparisons, to: 1: controls, 2: RD group, 3: SD group, 4: RSD.

There were no significant group differences on the nonverbal IQ test and the verbal WISC subtests (verbal short-term memory and vocabulary). Even though within the average range, both the RSD and SD groups showed significantly lower standard scores than controls on the symbol search task. On phonological awareness, Table 1 shows somewhat lower scores for the RD and the SD groups compared to controls, however, only the RSD group, who had the lowest score, was significantly different from all other groups. For RAN, the typical pattern emerged: the two groups with reading deficits (RD, RSD) showed lower performance than controls, while the SD group performed on the same level as controls. The ADHD score was largely comparable between control, RD and RSD group, but it was clearly higher (indicating more ADHD-symptoms reported by parents) for the SD group with a significant difference compared to controls.

### Cueing task

#### Analysis of raw RTs

Mean RTs were computed for each experimental condition for each participant and are reported in Table 2. The Kolmorgorov-Smirnov test for normality was not significant, *p* = .20, showing that RTs were normally distributed with a slight skewness to the right of .20 (*SE* = .18). Mean RTs were analyzed by a 4 (group: RSD, RD, SD, C) x 2 (cueing: valid vs. invalid) x 2 (position: left vs. right) ANOVA with group as between-subjects factor, and cueing and position as within-subject factors. Table 2 shows that the SD group had overall lower RTs than the three other groups, and indeed there was a significant baseline difference, *F*(3, 187) = 2.94, *p =* .03: post-hoc comparison with Bonferroni correction showed that the SD group responded significantly faster than the RSD group (418ms vs. 478ms, *p =* .03).

**Table 2:** Mean RTs (*M*) and standard deviations (*SD*) for each experimental condition of the visuo-spatial cueing paradigm in the four groups.

	C		RD		SD		RSD	
	*M*	*SD*	*M*	*SD*	*M*	*SD*	*M*	*SD*
Valid Left	441	110	487	94	408	101	478	133
Right	436	101	456	85	406	103	478	115
Invalid Left	477	121	501	109	436	134	486	121
Right	461	110	467	111	433	113	472	130
Valid combined	438	101	468	83	407	98	477	117
Invalid combined	469	110	485	104	433	118	479	118
Left combined	458	110	493	93	419	107	481	121
Right combined	447	99	460	92	418	104	475	113
Total	452	102	475	90	418	104	478	114

There was a main effect of cueing, *F*(1, 187) = 18.98, *p* < .001, η^2^ = .09, with lower RTs on valid than on invalid trials (449ms vs. 467ms). There was also a main effect of position, *F*(1, 187) = 12.26, *p <* .001, η^2^ = .06: RTs on the left (464ms) were higher than RTs on the right (451ms). The interaction cueing x group was also significant, *F*(3, 187) = 3.40, *p* = .02, η^2^ = .05 and the interaction position x group approached significance, *F*(3, 187) = 2.41, *p* = .07, η^2^ = .04. The remaining interactions were not significant (*Fs* between .20 and 1.10).

To explore the cueing x group interaction, paired-sample t-tests on valid vs. invalid RTs were run separately for each group, which showed a significant cueing effect for controls, *t*(65) = -4.90, *p <* .001 and the SD group, *t*(44) = -3.68, *p* = .001. The cueing effect closely failed to be significant in the RD group, *t*(27) = -1.78, *p* = .09 and was not significant in the RSD group, *t*(51) = -.25, *p* = .81.

To understand the position x group trend, paired-sample t-tests on left vs. right RTs were run for each group separately. The position effect, with higher RTs on the left, was significant for the RD group, *t*(27) = 4.44, *p* < .001 and for controls, *t*(65) = 2.00, *p* = .049, but it was not significant for the SD group, t(44) = .23, *p* = .82, and the RSD group, *t*(51) = .71, *p* = .48.

Thus, in summary the results of the raw score analysis largely confirmed earlier findings on cueing deficits in dyslexia: it appeared that indeed the two groups with reading deficits did not seem to profit from valid compared to invalid cues to the same extent as the non-impaired readers and therefore showed reduced cueing effects. Findings with respect to the position effect were less clear: a right-over-left advantage appeared to be particularly strong among the children with isolated reading deficits, while it was lacking for children with spelling problems (irrespective of an additional reading deficit).

However, we were concerned that the results might be influenced by the overall differences in RTs between the four groups, with SD children performing faster than the other groups. In order to control for these baseline differences, we decided to rerun the analysis based on individually *z*-transformed data.

#### Analysis of *z*-transformed RTs

All RTs were transformed into *z*-scores referring to the individual mean of each participant [52] and then mean *z*-transformed RTs (zRTs) were computed for each experimental condition, with lower *z*-scores representing lower RTs. Furthermore, we calculated a cueing index and a position index for each participant. The cueing index represents the difference between invalid and valid zRTs. Positive values of the cueing index indicate that—as expected—responses on valid trials were faster than on invalid trials. A cueing index of zero thus means that the participant responded equally fast to valid and invalid trials and did not show any effect of cueing. The position index represents the difference between left and right zRTs, with positive values indicating higher zRTs on the left, and negative values indicating higher zRTs on the right.

Due to the *z*-standardization, the main effect of group was not significant, *F*(3, 187) = .57, *p =* .64. There was a significant cueing effect *F*(1, 187) = 27.81, *p* < .001, η^2^ = .13, with lower zRTs for valid (-.07) than invalid trials (.10). The position effect was also significant, *F*(1, 187) = 12.36, *p* = .001, η^2^ = .06, with overall lower zRTs on the right (-.04) than on the left (.07). Again, group interacted with cueing condition *F*(3, 187) = 2.87, *p* = .04, η^2^ = .04 and the group x position interaction was also significant, *F*(3, 187) = 2.86, *p* = .04, η^2^ = .04.

**Fig 2:**
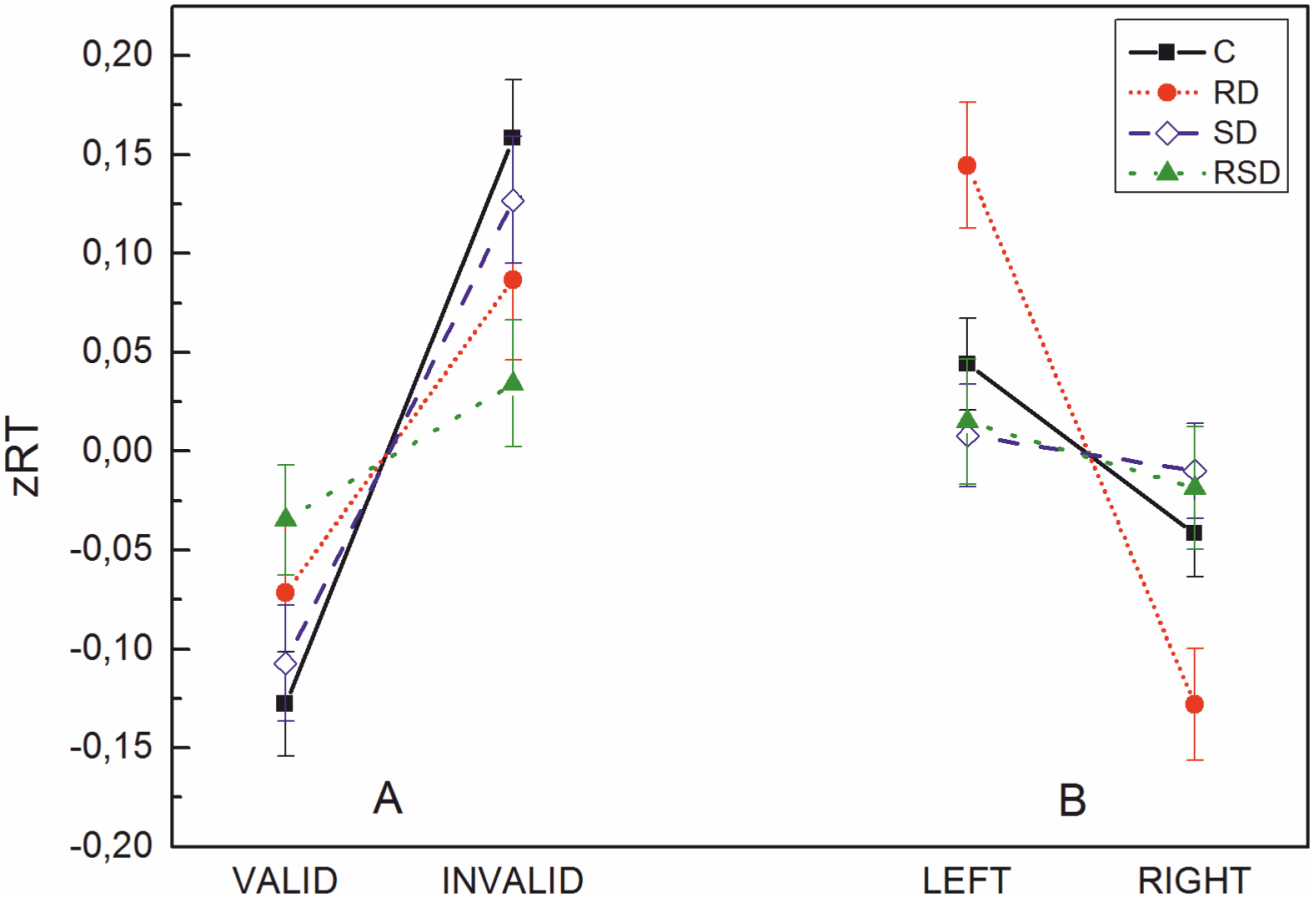
zRTs for each group (C, RD, SD, RSD) for valid vs. invalid (A) and left vs. right (B). Bars represent standard errors.

However, the pattern of interactions was different to the raw score analysis: Fig 2A presents the cueing effect combined across left and right positions. Paired-sample t-tests on valid vs. invalid mean zRTs separately for each group showed a significant cueing effect for the RD group (zRT difference = .16), *t*(27) = -2.07, *p* = .048, for the SD group (zRTs difference = .23), *t*(44) = -3.84, *p* < .001 and for controls (zRT difference = .29), *t*(65) = -5.18, *p* < .001; whereas the cueing effect was not significant in the RSD group (zRT difference = .07), *t*(51) = -1.16, *p* = .25. The cueing effect in the RD-group seemed somehow smaller than in the other two groups (SD and controls). However, a one-way ANOVA on the cueing index with group (controls, RD, and SD) as the between-subjects factor revealed no significant difference F(2, 138) = .89, *p* = .41.

Fig 2B presents the position effect for the four groups, combined across valid and invalid trials. Paired-sample t-tests on left vs. right mean zRTs separately for each group showed a significant position effect for the RD group (zRT difference = .27), *t*(27) = 4.56, *p <* .001 and a trend for controls (zRT difference = .09), *t*(65) = 1.92, *p* = .06. The position effect was not significant for the SD group (zRT difference = .02), *t*(44) = .36, *p* = .72) and the RSD group (zRT difference = .03), *t*(51) = .54, *p* = .59. An independent samples t-test on the position index showed that the left-right difference on zRTs was significantly larger in the RD group compared to controls *t*(92) = 2.38, *p* = .02.

In summary, the *z*-score analyses revealed an important difference to the raw score analysis, as the RD group now shows a significant cueing effect, which was only marginal on raw RTs. Only the RSD group´s findings were in line with the prediction of a visual attention deficit in dyslexia: Indeed, they did not seem to be influenced by the presentation of a visual cue.

#### Controlling for ADHD-score

In order to investigate the impact of general attention on children´s performance, the same analysis (group x cueing x position ANOVA) was repeated introducing the individual ADHD score as a covariate. It turned out that the ADHD score was a non-significant covariate at the between-subject level *F*(1, 186) = 2.70, *p* = .10 as well as at the within-subject level (*Fs* between .18 and 2.60).

#### Group analyses based on sublexical NW reading

**Fig 3:**
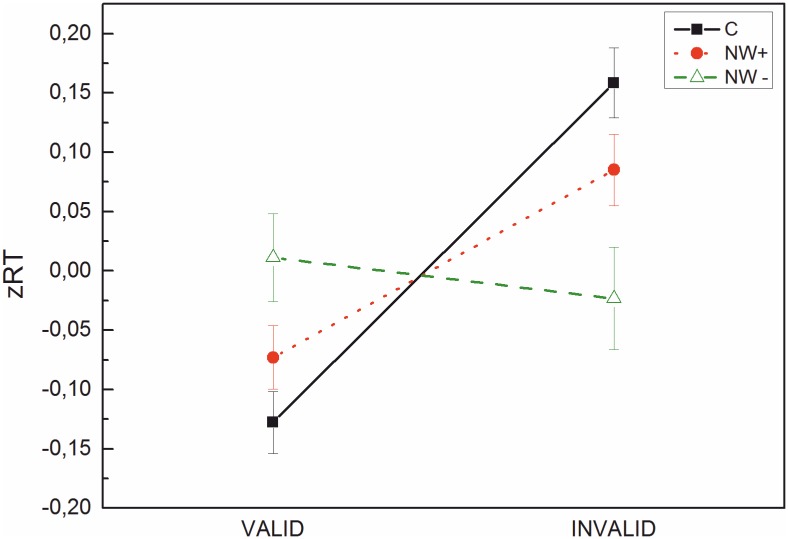
zRTs for poor nonword readers (NW-), adequate nonword readers (NW+) and controls on valid and invalid trials. Bars represent standard errors.

In the next step, we specifically tested the hypothesis that the cueing deficit appears only in children with particular problems in nonword decoding. Following an analysis presented by [11], we classified reading disabled children as “poor decoders” (NW-) if their nonword reading raw score was ≤ 1.5 standard deviations below the mean (*n* = 24). This group included 7 of the 28 RD-children (25%) and 17 of the 52 RSD-children (33%).The remaining children with a reading deficit but nonword performance not more than 1.5 standard deviations below mean were classified as “adequate decoders” (NW+; *n* = 56). These two groups were compared with the control group of children with typical development (*n* = 66). Mean zRTs for valid and invalid trials are presented in Fig 3. A group (NW-; NW+; control) x cueing x position ANOVA showed significant effects of cueing, *F*(1, 143) = 8.94, *p* = .003, η^2^ = .06, and position, *F*(1, 143) = 8.43, *p* = .004, η^2^ = .06, and a significant cueing x group interaction *F*(2, 143) = 5.65, *p* = .004, η^2^ = .07. The remaining interactions as well as the between-subjects factor group were not significant (*Fs* between .001 and 2.15). To explore the cueing x group interaction, follow-up paired-sample t-tests were conducted on the two NW groups (for the control group, the cueing effect was already established above). A significant cueing effect was present for NW+ (zRTs difference = .16), *t*(55) = -2.78, *p* = .01 but not for NW- (zRTs difference = .03), *t*(23) = .44, *p* = .67.

#### Group analysis based on lexical spelling

**Fig 4:**
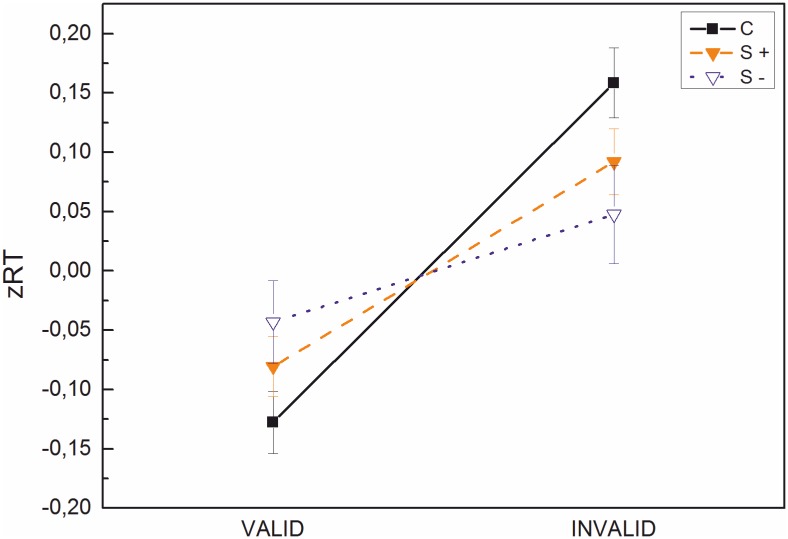
zRTs for poor spellers (S-), adequate spellers (S+) and controls on valid and invalid trials. Bars represent standard errors.

A similar analysis was run based on three groups that differed in orthographic spelling as an indicator of lexical processing. Children were classified as S- if their spelling raw score was ≤ -1.5 SD from the mean (*n* = 32). This group included 8 of the 45 SD-children (18%) and 24 of the 52 RSD-children (46%). The remaining children of these two groups with spelling scores > -1.5 SD from the mean were classified as S+ (*n* = 65). These two groups were compared to controls (*n* = 66). Mean zRTs for valid and invalid trials are presented in Fig 4. A group (S-; S+; C) x cueing x position ANOVA showed a significant cueing effect *F*(1, 160) = 23.43, *p* < .001, η^2^ = .13 and a trend towards significance for the cueing x group interaction *F*(2, 160) = 2.54, *p* = .08, η^2^ = .03 and the cueing x position interaction *F*(1, 160) = 3.54, *p* = .06, η^2^ = .02. The remaining effects and interactions were not significant (*Fs* between .15 and 2.0).

To explore the marginally significant cueing x group interaction, paired-sample t-tests were conducted on the two spelling level groups, separately. A significant cueing effect was present for S+ (zRT difference = .17), *t*(64) = -3.27, *p* = .002, but not for S-, (zRT difference = .09), *t*(31) = -1.20, *p* = .24).

#### Group comparison based on cueing index

Next, we wanted to determine how many children of our sample were unable to make efficient use of the visual cues. In order to estimate a threshold for a cueing index that was different from zero, we specified a confidence interval based on the distribution of our large sample: We split children´s RTs on valid trials into two halves (odd vs. even correct trials) and we calculated a mean score difference for each participant. The mean difference score was expected not to be different from zero, which was confirmed by a one sample t-test against zero, *t*(190) = .06, *p* = .96. The observed mean difference score of .002 and its 95% CI [-.07, .07] were taken as statistical operationalization of a theoretical 0 value: the upper bound of the confidence interval of .07 was used as a cutoff between participants who show positive cueing, and those who show absent/negative cueing. That way, 121 children with a cueing index above .07 were classified as “positive cueing”, whereas 70 children with a cueing index equal or below .07 were classified as “absent/negative cueing”.

The “absent/negative cueing” group included 21 of the 66 control children (32%), 10 of the 28 RD-children (36%), 14 of the 45 SD-children (31%), and 25 of the 52 RSD-children (48%). Thus, the cueing groups were distributed fairly equally across the four literacy groups. Importantly, about one third of the control sample did not show a clear cueing effect.

Table 3 presents the literacy and cognitive variables for the two cueing index groups. A trend for significant group differences was present for sentence and nonword reading, with the positive cueing group showing better performance in these measures than the absent/negative cueing group, and for digit span, with the absent/negative cueing group having a slightly higher score.

**Table 3:** Means (*M*) and standard deviations (*SD*) for the literacy and cognitive measures in the two cueing index-based groups.

	Positive cueing *n* = 121		Absent / Negative cueing *n* = 70		*t*	*p*
	*M*	*SD*	*M*	*SD*		
Sentence reading (SLS)						
percentile	36.23	22.84	30.76	24.97	-1.53	0.13
raw score	30.48	7.75	28.26	9.17	-1.69	0.09
Word reading (SLRT-II)						
percentile	34.09	22.43	30.95	24.99	-0.87	0.39
raw score	52.21	15.96	49.47	18.30	-1.04	0.30
Pseudoword reading (SLRT-II)						
percentile	36.84	24.45	31.44	24.84	-1.46	0.14
raw score	33.87	8.70	31.47	9.27	-1.79	0.07
Spelling (DRT-3)						
percentile	32.31	22.72	28.91	22.37	-1.00	0.32
raw score	25.52	8.03	27.16	8.42	1.33	0.19
Non Verbal IQ (CFT-20)	105.70	12.51	106.89	11.41	0.65	0.52
WISC-IV (standard score)						
vocabulary	12.02	3.25	12.37	3.13	0.72	0.47
digitspan	9.82	2.13	10.46	2.49	1.87	0.06
symbol search	11.24	2.39	11.21	2.05	-0.07	0.94
Phonological awareness (% correct)	0.74	0.17	0.76	0.14	0.89	0.38
RAN numbers	1.98	0.39	1.91	0.45	-1.16	0.25
ADHD questionnaire	0.51	0.35	0.52	0.34	0.31	0.75

#### Group comparison based on position index

We identified 107 children with a positive position index (right-over-left advantage) and 84 with a negative position index (left-over-right advantage). The positive position index group included 38 out of 66 control children (57%), 24 out of 28 RD-children (85%), 20 out of 45 SD-children (44%) and 25 out of 52 RSD-children (48%). A Chi-Square Test yielded a significant association between literacy group and position index group, χ^2^(3; *N* = 191) = 13.87; *p* = .003. When we reran the Chi-Square test without the RD group, it was no longer significant, χ^2^(2; *N* = 163) = 2.09, *p* = .35, confirming that the distribution of a right-over–left advantage effect was clearly higher in the RD than the three other groups, where it was fairly comparable.

Independent-samples t-tests on literacy and cognitive variables revealed only two significant differences: Nonverbal IQ was higher in the positive compared to the negative position index group (108 vs 104, *t*(189) = 2.42, *p* = .02) and similarly, number of correctly spelled words was higher in the positive than the negative position index group (19 vs. 16, *t*(187) = 2.55, *p* = .01).

## Discussion

The present study investigated visuo-spatial attention abilities in a large sample of children with isolated or combined deficits in reading and/or spelling and age-matched controls. Participants were carefully selected based on their performance on standardized tests of reading and spelling and presented with a standard visual cueing paradigm, which was modelled after the paradigm used by [11]. Eye-tracking was applied in order to ensure that children followed the instruction to look at the fixation cross when cues and targets were presented in the periphery of their visual field. This procedure allowed us to rule out the presence of eye-movements during the task and ensured that only attention orienting could explain the cueing or position effects.

When we analyzed children´s raw RTs, our findings seemed to largely confirm the visuo-spatial attention deficit hypothesis of dyslexia [9]: While children with age-adequate reading performance seemed to process the cue as expected and showed faster responses to valid than to invalid trials, both groups of poor readers showed reduced cueing effects. Furthermore, no association of the cueing effect was observed with children´s spelling skills as the group with isolated spelling deficits did seem to profit from valid compared to invalid cueing of the target. However, this group also showed relatively fast overall response times while the RSD group showed relatively slow overall response times. Obviously, such baseline differences make it difficult to interpret any non-orthogonal interactions involving groups. Note that some earlier studies on visual cueing in dyslexia also reported baseline differences [6,9], which were not controlled for in further analyses. In the present study we controlled for the baseline differences by rerunning the analysis based on individually *z*-standardized cueing effects. In this second analysis, the pattern of findings appeared to be different: Now the only group that did not show a significant cueing effect was the group of children with poor performance in reading as well as spelling (RSD), while the RD and SD groups did profit from valid compared to invalid cueing. Thus, quite surprisingly, relating the cueing effect to children´s individual overall response times did not only impact on the interaction of the cueing effect with group, but helped to identify standard cueing performance in the RD-group. Note that our RSD group showed word and nonword reading performance that was comparable to the RD group. Thus, it is not the case that the RD group had a milder reading problem than the RSD group.

Focused visuo-spatial attention is supposed to be particularly important for grapheme parsing during sublexical reading. Interestingly, when we specifically tested for associations with sublexical reading by comparing children with a nonword reading deficit with poor readers who showed acceptable nonword reading efficiency and the control group, our findings confirmed earlier evidence [10–12] in that it was the poor nonword readers who did not seem to profit from a valid visual cue. Again, it is important to point out that the two reading disabled groups were matched on nonword reading and the percentage of poor nonword readers in the RD and RSD groups was about equal (25 and 33%). Still, as the RD group was clearly smaller than the RSD group, most children with poor nonword reading skills also experienced severe problems with spelling. When we ran an analogous analysis testing for specific associations of a cueing deficit with lexical spelling, the children with the lowest spelling scores did not show a cueing effect. It is possible that the association with spelling shows the involvement of visuo-spatial attention in building up orthographic knowledge as it provides precise positional information of letter identities within words during phonological decoding [29].

The current study could also extends our understanding of an unduly large right-over-left advantage in dyslexia [23–26]: We found a particularly marked right-over-left advantage among RD children, while the SD and RSD groups did not show a position effect. It has been claimed that the position effect may be associated with practicing left-to-right visual processing during reading [35,53,54], but see [55]. In the current study, this seems unlikely as the two groups of poor readers were matched on reading, but still showed different position effects. This explanation is also not in line with the finding that the position effect was larger in the RD than in the control group, although the latter should have more reading experience. A tentative explanation for the strong position effect among poor readers who nevertheless were able to develop age-adequate spelling is that attention in the right visual field may be helpful to perceive and store orthographic markers that occur to the right of the first fixation on a word. As German words (particularly verbs) usually end with grammatically important inflectional morphemes, attention to the right visual field may be relevant in order to build up orthographic representations.

Our findings did not corroborate our concerns that deficits in visual attention might be part of a broader range of attentional problems in the ADHD-spectrum rather than a causal factor in dyslexia. Note that—as in other studies—we excluded children with an ADHD-diagnosis from participation. Still, there were group differences in parents´ ratings of ADHD-symptoms. Such subclinical differences in attention, impulsivity, and hyperactivity are often ignored in studies on learning disorders, although they may have an important impact, particularly on tasks that are not always very engaging and require high levels of attention [42,56]. However, we found that the parental ADHD-score did not interact with the *z*-standardized cueing and position effects. Still, future studies may want to assess attention more closely with neuropsychological tests, which are probably more sensitive to differences in attentional profiles than a parental questionnaire.

An important contribution of the current study is that our sample size allowed us to investigate the prevalence of visuo-attentional deficits among children with typical development of written language processing. In order to achieve this, we computed a cueing index. A positive cueing index expresses the size of the advantage of valid compared to invalid cues in responding to the target. Interestingly, about 60% of our participants showed a cueing index that was significantly above zero. A cueing index around zero means that the cue was not sufficiently processed in order to influence participants´ response to targets. A negative cueing index, meaning that targets preceded by an invalid cue were detected faster than targets preceded by a valid cue, is difficult to explain. It might be considered as resulting from inhibition of return, a mechanism that has been proposed to facilitate visual search because it avoids the re-inspection of previously explored locations due to attentional [14,57] or sensory processes [58,59]. However, this explanation is unlikely in the current experimental setting, where only peripheral cues were presented and manual reaction times were measured. We used very short presentation times (SOA = 100ms), whereas the inhibition of return was shown to appear on similar exogenous paradigms for SOAs higher than 300ms [58,59].

When we compared participants with positive cueing index (indicating efficient processing of cues) vs. absent/negative cueing index (indicating inefficient cue-processing), we found lower performance in nonword reading and sentence reading in the group with inefficient cue-processing. We found that 48% of the RSD and 36% of the RD group showed compromised visual attention. These percentages are roughly in line with Carroll et al., who reported prevalence rates of 23 to 45% for a range of potential cognitive factors significantly related to later reading problems. But while [5] found relatively low prevalence rates of about 10% for these cognitive deficits among typically developing readers, we observed that about one third of the control group also had marked problems to make efficient use of the visual cue in our paradigm. If a cueing deficit is assumed to cause problems in graphemic parsing in the context of reading development, the high rate of children with a supposedly causal deficit who do not experience any impairments in reading and/or spelling is at least surprising and the factors under which this deficit has or does not have a negative effect on children´s written language processing should be specified in terms of a multi-deficit theory of dyslexia [6].

In the current study, reasonable sample size for different profiles of reading and spelling deficits was given preference over a more fine-grained assessment of visuo-spatial attention. In order to keep task duration reasonably short for our young participants, the number of items was relatively small and we did not experimentally vary SOAs, eccentricity or size of the visual cues as has been done in other studies [24,60]. Our item set also did not include neutral items (i.e., a target without a preceding cue) so that it is unclear whether the cueing effect in our study results from facilitation of target identification in valid items or from lack of inhibition of the cue in invalid trials. Indeed, the aim of the study was not to further specify the type of deficits that can exist in visuo-spatial attention. The important contribution of our large sample is that the variability of spatial-attentional profiles becomes evident: While one group of poor readers, who also had spelling problems, showed evidence for a cueing deficit, another group with equally poor reading skills but intact spelling showed intact cueing, but an exceptionally strong right-over-left position effect. Finally, a group of children with isolated spelling deficits showed the same cueing performance as typically developing controls, but while controls exhibited a marginally significant position effect, this effect was lacking among the poor spellers. It is possible, that these group differences reflect systematic differences in attentional profiles. We might speculate that the lacking cueing effect in the RSD-group indicates problems to orient attention quickly towards visual cues, perhaps because of an overly broad attentional window, which may hamper focusing. On the other hand, the unduly strong position effect in accordance with a pretty intact cueing performance in the RD group could be interpreted as a reduced attentional window, which is somewhat shifted to the right due to reading experience, but can adequately orient towards incoming visual information. However, as our study is cross-sectional, we cannot make any claims on causality. And we also need to consider that there is variability within each subsample with only between 52 and 69% of children showing a clear cueing effect and 44 to 85% showing a position effect. This variability probably also explains inconsistencies among earlier studies. It will be important that future studies assess reading as well as spelling profiles to better understand differences in visuo-spatial attention performance among children with different literacy profiles.

Our findings show that both, visuo-spatial attention and written language processing are multifaceted constructs and their interactions during reading development are complex. It is without question that a reasonable amount of visuo-spatial attention is necessary for written language processing and deficiencies in this domain are likely to constitute one of the risk factors in a multifactorial model of dyslexia [1]. It will be important to specify the exact role of visuo-spatial processing and its subcomponents within the risk and protective factors as well as potential causal links with the developing reading system.
